# Role of EBNA-3 Family Proteins in EBV Associated B-cell Lymphomagenesis

**DOI:** 10.3389/fmicb.2016.00457

**Published:** 2016-04-07

**Authors:** Shaoni Bhattacharjee, Shatadru Ghosh Roy, Priyanka Bose, Abhik Saha

**Affiliations:** Department of Biological Sciences, Presidency UniversityKolkata, India

**Keywords:** EBV, B-cell lymphoma, LCLs, EBNA-3 proteins, EBNA-3C, EBNA-3A, EBNA-3B

## Abstract

Epstein-Barr virus (EBV) is highly ubiquitous in human population and establishes a lifelong asymptomatic infection within the infected host unless the immune system is compromised. Following initial infection in the oropharyngeal epithelial cells, EBV primarily infects naive B-lymphocytes and develops a number of B-cell lymphomas particularly in immune-deficient individuals. *In vitro*, EBV can also infect and subsequently transform quiescent B-lymphocytes into continuously proliferating lymphoblastoid cell lines (LCLs) resembling EBV-induced lymphoproliferative disorders in which a subset of latent transcripts are detected. Genetic studies revealed that EBNA-3 family comprising of three adjacent genes in the viral genome—EBNA-3A and -3C, but not -3B, are critical for B-cell transformation. Nevertheless, all three proteins appear to significantly contribute to maintain the overall proliferation and viability of transformed cells, suggesting a critical role in lymphoma development. Apart from functioning as important viral transcriptional regulators, EBNA-3 proteins associate with many cellular proteins in different signaling networks, providing a suitable platform for lifelong survival of the virus and concurrent lymphoma development in the infected host. The chapter describes the function of each these EBV nuclear antigen 3 proteins employed by the virus as a means to understand viral pathogenesis of several EBV-associated B-cell malignancies.

## Introduction

Epstein-Barr virus (EBV) nuclear antigen, EBNA-3, comprising of three closely related proteins namely EBNA-3A, -3B, and -3C, was suggested to be generated by a series of gene duplication events during gammaherpesvirus evolution as they share limited but significant amino acid (aa) sequence homology (Saha and Robertson, 2013). Interestingly, unlike of the other viral genes there are no known viral homologs in other closely related primate lymphocryptoviruses. They share a similar gene structure with a shorter 5′- and a longer 3′-exons arranged in a tandem array in the EBV episome. All EBNA-3 transcripts are alternatively spliced from very long mRNAs initiated at the latency C-promoter (Cp), which is active in EBV transformed lymphoblastoid cell lines (LCLs) but blocked in several EBV-associated cancers through hypermethylation [reviewed in (Robertson et al., 1995; Saha and Robertson, 2013; Allday et al., 2015)]. Although, EBNA-3 proteins demonstrated extensive redundant biological functions, genetic studies using recombinant viruses revealed that only EBNA-3A and -3C but not -3B are essential for B-cell transformation *in vitro* (Maruo et al., 2005, 2006, 2011; Saha and Robertson, 2013).

### Initial detection of EBNA-3 proteins in EBV infected B-cells

The EBNA-3 proteins or rather EBNA-3 was initially identified as an extra 142-kDa band along with other previously identified latent proteins—EBNA-1, EBNA-2 and LMP-1, in latently infected B-cell cultures using EBV positive patient's sera (Hennessy et al., 1985). Initial experiments demonstrated that EBNA-3 was generated from the *B**am*HI digested fragment (named as ‘E’) Rightward open reading Frame 1 (BERF1) of EBV episome (reviewed in Hennessy et al., 1986; Saha and Robertson, 2013). Interestingly, a number of human sera were tested demonstrated slightly higher molecular weights than EBNA-3 that was previously detected, signifying that EBNA-3 might be composed of several related proteins within same family in EBV transformed B-lymphocytes. Following studies demonstrated that indeed EBNA-3 was composed of three proteins—in addition to the initially identified BERF1 fragment encoding EBNA-3A, *Bam*HI BERF2b fragment expressed a 165 kDa protein named EBNA-3B (or EBNA-4) and the most rightward short and long *Bam*HI E fragments (BERF3 and BERF4) expressed a 155 kDa protein named EBNA-3C (or EBNA-6) (Petti and Kieff, 1988; Petti et al., 1988; Saha and Robertson, 2013). Interestingly, EBNA-3C was detected nearly in all EBV positive B-cells except Raji as it was found later that there was a deletion of the BERF4 segment of the EBV episome in this cell line (reviewed in Saha and Robertson, 2013).

### EBNA-3 proteins are restricted to nucleus

Sub-cellular localization of all EBNA-3 proteins was found to be restricted to the nucleus using affinity chromatography purified human anti-sera. Subsequent studies in order to delineate the functional domains of EBNA-3A demonstrated that the aa residues 147-157 contain a nuclear localization sequence (NLS). Computational prediction analyses in combination with molecular cell biology experiments using GFP-tagged constructs and site directed mutagenesis further confirmed that EBNA-3A contains 5, EBNA-3B contains 2 and EBNA-3C contains 3 functional NLSs (Krauer K. et al., 2004; Buck et al., 2006; Burgess et al., 2006).

### EBNA-3 expression is restricted to latency III program

The characterized expression pattern of latent genes in EBV-transformed LCLs is known as “latency-III program.” This pattern is also observed in most of the post-transplant and immune-compromized patients' associated EBV positive lymphomas. In this program, all the latent genes are expressed—a subset of 12 transcripts, which include six nuclear proteins EBNA-1, EBNA-2, EBNA-3A, EBNA-3B, EBNA-3C, and EBNA-LP, three membrane proteins LMP-1, LMP-2A, and LMP-2B along with three non-coding unpolyadenylated transcripts EBER-1 and EBER-2, and BARTs (Saha et al., 2010; Saha and Robertson, 2011a). Although infected cells contain only a few copies of EBNA-3 transcripts, the EBNA-3 protein products are relatively stable *in vivo*. It has been demonstrated that the latency-III program can be simultaneously developed in conjunction with acute EBV infection in nascent B-cells prior to the proper establishment of latently infected memory B-cells, which serves as a latent reservoir of virus particles and subject to temporal activation of different latency programs (Young and Rickinson, 2004).

### EBNA-3A and -3C, but not -3B, are essential for *in vitro* B-cell transformation

A series of experiments using genetically engineered viruses confirmed that only EBNA-3B of EBNA-3 proteins can be expendable for EBV-mediated B-cell growth transformation *in vitro* (Chen et al., 2005; Maruo et al., 2006, 2011). Initial molecular biology experiments with recombinant EBV containing stop codons inserted into the ORFs of other family members established that both EBNA-3A and -3C are indispensable for B-cell growth transformation *in vitro* (Tomkinson et al., 1993). Whilst recombinant virus encoding an EBNA-3A protein with a stop codon inserted after aa 302 lost its ability to transform naive B-cell, a parallel expression of wild-type EBNA-3A was typically shown to restore the transforming ability of the mutant virus. In corroboration to this, kempkes et al. also demonstrated the importance of EBNA-3A in maintaining EBV transformed B-cell outgrowth using a 71 kbp of EBV episome termed as mini-EBV with a single C residue deleted at EBNA-3A ORF (Kempkes et al., 1995). Similar studies using recombinant virus containing a stop codon at aa 365 in EBNA-3C ORF demonstrated its essentiality in B-cell transformation, whereas recombinant virus encoding an EBNA-3B protein with a stop codon inserted after aa 109 was unable to show any difference in B-cell transformation when compared to wild-type virus (Tomkinson and Kieff, 1992; Tomkinson et al., 1993). In addition, recently EBNA-3B was rather described as a tumor suppressor protein in comparison to an intense oncogenic activities exerted by the other two family members EBNA-3A and -3C (reviewed in Allday et al., 2015).

Although, EBNA-3B was shown to dispensable for EBV-induced B-cell growth transformation, fascinatingly all the EBNA-3 proteins were recognized as major antigenic targets for cytotoxic T-cell (CTL) responses against EBV-transformed B-lymphocytes (Murray et al., 1992), signifying the importance of each of the EBNA-3 members in primary EBV-infection and subsequent B-cell lymphoma development. Herein we aim to summarize the work of more than two decades focussing on EBNA-3 proteins in terms of their molecular interactions with multiple host cellular networks.

## Transcriptional regulation

### EBNA-3 proteins are non-DNA binding potent transcriptional regulators

Regardless of their minimal structural resemblance, all EBNA-3 proteins function as transcriptional regulatory proteins through interacting with numerous cellular DNA binding proteins and other accessory transcription factors, instead of directly bind to the DNA (Table 1).

**Table 1:** **EBNA-3 interacting cellular partners**.

**Interacting proteins**	**Deregulated function**	**References**
**EBNA-3A**		
RBP-Jk	Recruits RBP-Jκ activity to regulate both viral and cellular gene transcription	Robertson et al., 1996; Maruo et al., 2005; Calderwood et al., 2011
CtBP	Recruits CtBP to regulate gene transcription, for example p16^INK4A^	Hickabottom et al., 2002; Skalska et al., 2010
Chk2	Releases G2/M cell-cycle block	Krauer K. G. et al., 2004b
WDR48, WDR20, and USP46/UP12	Forms a deubquitylation complex consisting of WDR48, WDR20, and USP46/USP12), possibly required to maintain LCL outgrowth	Ohashi et al., 2015
MIZ-1	Blocks the interaction between MIZ-1 and nucleophosmin and overall inhibits p15^INK4B^ expression	Bazot et al., 2014
20S proteasome	Not yet described	Touitou et al., 2005
Chaperones	Induces all of the factors necessary for an active Hsp70 chaperone complex including Hsp70, Hsp70B/B′, Bag3, and DNAJA1/Hsp40	Young et al., 2008
XAP-2	Increased nuclear localization; however, precise function has not yet been described	Kashuba et al., 2000
TCP-1	Not yet described	Kashuba et al., 1999
AhR	Enhances ligand dependent transactivation	Kashuba et al., 2006
UK/UPRT	Increased nuclear localization; however, precise function has not yet been described	Kashuba et al., 2002
**EBNA-3B**		
RBP-Jκ	Recruits RBP-Jκ activity to regulate both viral and cellular gene transcription	Robertson et al., 1996
Cyclin A	Not yet described	Knight and Robertson, 2004
WDR48, WDR20, and USP46/UP12	Forms a deubquitylation complex consisting of WDR48, WDR20, and USP46/USP12)	Ohashi et al., 2015
20S proteasome	Not yet described	Touitou et al., 2005
**EBNA-3C**		
RBP-Jκ	Recruits RBP-Jκ activity to regulate both viral and cellular gene transcription	Robertson et al., 1996; Lee et al., 2009; Calderwood et al., 2011; Kalchschmidt et al., 2016
CtBP	Recruits CtBP to regulate gene transcription, for example p16^INK4A^	Touitou et al., 2001; Lee et al., 2009
Prothymosin-α	Recruits prothymosin-α along with p300 to regulate gene transcription	Cotter and Robertson, 2000
p300	Recruits p300 activity to regulate cellular gene transcription	Subramanian et al., 2002a
Gemin3/DDX20	Stabilizes and promotes a complex formation with p53 and thereby negatively affecting p53 transcriptional activity	Cai et al., 2011
HDAC1 and HDAC2	Recruits HDAC1/2 activity to regulate cellular gene transcription	Radkov et al., 1999; Knight et al., 2003
mSin3A and NCoR	Functions in a complex with mSin3A and NCoR and represses transcription, for example p16^INK4A^	Knight et al., 2003; Jiang et al., 2014
Spi1/PU1	Recruits to regulate both viral and cellular gene transcription	Zhao and Sample, 2000
Nm23-H1	Modulates intrinsic transcriptional and anti-metastatic activities	Subramanian et al., 2001
20S proteasome	Not yet described	Touitou et al., 2005
Cyclin A	Enhances Cyclin A/CDK2 kiase activity	Knight and Robertson, 2004; Knight et al., 2004
Cyclin E	Not yet described	Knight et al., 2004
Cyclin D1	Enhances the kinase activity of Cyclin D1/CDK6 which enables subsequent ubiquitination and degradation of pRb	Knight et al., 2004; Saha et al., 2011b
GSK3β	Increases Cyclin D1 nuclear localization by blocking GSK3β activity	Saha et al., 2011b
p53	Blocks p53 mediated transcriptional and apoptotic activities	Saha et al., 2009, 2011a; Yi et al., 2009
Mdm2	Enhances the intrinsic ubiquitin ligase activity of Mdm2 toward p53	Saha et al., 2009
ING4 and ING5	Suppresses both ING4 and ING5 assisted p53 transcriptional activity	Saha et al., 2011a
E2F1	Represses E2F1 transcriptional activity in response to DNA damage signals	Saha et al., 2012
pRb	Facilitates pRb degradation in an ubiquitin-proteasome manner	Knight et al., 2005a
Chk2	Releases the G2/M checkpoint block of cell-cycle	Choudhuri et al., 2007; Nikitin et al., 2010
Gadd34	Activates the upstream component of the UPR (eIF2α-phosphorylation) and blocks downstream UPR events (XBP1 activation and ATF6 cleavage)	Garrido et al., 2009
SCF^Skp2^	Recruits of SCF^Skp2^ E3 ligase activity to facilitate degradation of p27^KIP1^ and pRb	Knight et al., 2005a,b
c-Myc	Stabilizes and enhances transcription	Bajaj et al., 2008
Sumo1/3	Recruits SUMO-1/3 for EBNA-2 mediated transctivation	Rosendorff et al., 2004
MRPS18-2	Releases E2F1 and thereby facilitates G1-S transition of the cell-cycle	Kashuba et al., 2008
Aurora kinase B	Negatively regulates p53 and pRb activities through stabilizing Aurora kinase B activity	Jha et al., 2013, 2015
IRF4/8	Stabilizes IRF4 and dowregulates IRF8; Recruits for modulating cellular gene transcription	Banerjee et al., 2013; Jiang et al., 2014
H2AX	Destabilizes H2AX and restricts H2AX expression into nucleus	Nikitin et al., 2010; Jha et al., 2014
USP46/USP12	Recruits USP46/USP12 dubiquitination activity to regulate p14^ARF^ transcription	Ohashi et al., 2015
p73	Blocks p73 mediated apoptosis	Sahu et al., 2014

### RBP-Jk—a common mediator of EBNA-3 mediated transcriptional repression

A large number of interacting proteins for EBNA-3 have been identified and subsequently suggested to be crucial for EBV induced B-cell transformation. Of these, RBP-Jk (or CBF1), a downstream regulator of Notch signaling pathway, was the first established transcription factor required for LCL growth (Lee et al., 2009; Maruo et al., 2009). The initial clue that EBNA-3 proteins can function as transcriptional regulators was derived from the seminal observation that all EBNA-3 proteins share a common binding site of RBP-Jk (Waltzer et al., 1996; Saha and Robertson, 2013). EBNA-2, another essential latent antigen for *in vitro* B-cell transformation, was also found to act as a non-DNA binding transcriptional activator for both viral (LMP-1 and LMP-2) and cellular genes (CD23) through recruiting RBP-Jk activity (Wang et al., 1991; Waltzer et al., 1996). Interestingly, all three EBNA-3 proteins can antagonize EBNA-2 induced transcriptional activation through competing with RBP-Jk binding (Waltzer et al., 1996). Using simple reporter assays it has later been revealed that all EBNA-3 proteins are strong transcriptional repressors. Most of the earlier work on EBNA-3 mediated transcriptional repression was particularly focussed on EBNA-3C protein. Two putative repressive domains for EBNA-3C have been identified—one lies at aa 280-525 represents as strong repressor domain and another lies at aa 580-992 represents as relatively weaker repressive domain (Subramanian et al., 2002b; West, 2006; Saha and Robertson, 2013). As similar to EBNA-3C, an inherent transcriptional repression domain of EBNA-3A was mapped within aa 100-364. Additionally, a RBP-Jk-independent repression domain of EBNA-3A was also mapped to aa 524-666. For example, EBNA-3A can efficiently suppress EBNA-2 mediated activation from the EBV Cp promoter, while the repressive effect was not observed once the RBP-Jk binding site in the promoter region was absent (Cludts and Farrell, 1998). Given the viral Cp promoter regulates the transcription of all EBNA genes, it can be easily speculated that the EBNA-3 proteins might have an auto-regulatory function in order to control their own expression in latently infected B-lymphocytes.

All the three EBNA-3 proteins were shown to interact with RBP-Jk through their homology sequences located at the N-terminal region (Robertson et al., 1996; Waltzer et al., 1996). In case of EBNA-3A, the binding domain was mapped at aa 1-138, in which aa 125-138 were found to be important (Bourillot et al., 1998). Interestingly, several other groups subsequently mapped the RBP-Jk binding domain spanning different aa residues located either at 173-223 or 224-566 of EBNA-3A (Zhao et al., 1996). The RBP-Jk binding domain of EBNA-3B was mapped at the conserved N-terminus region spanning aa 1-311 (Robertson et al., 1996). Likewise EBNA-3C binding domain to RBP-Jk has also been mapped at the N-terminal region spanning amino acid residues 1-183 (Robertson et al., 1996). Interestingly, all the EBNA-3 proteins were shown to interact with a RBP-Jk homolog, RBP-2N (Krauer et al., 1996). However, the functional significance of these interactions is not yet explored.

More recently, it has been clearly demonstrated the interaction between EBNA-3 proteins with RBP-Jk is vital for maintaining LCL growth, where EBNA-3 mediated suppression of EBNA-2 induced Cp promoter might play an important role (Maruo et al., 2005, 2009). LCLs established with recombinant viruses that have deleted portions of both EBNA-3A and -3C binding sites for RBP-Jk were unable to grow, whereas ectopic expression of individual wild-type cDNAs maintained the proliferation (Maruo et al., 2005, 2009). However, whether two seemingly different functions - EBNA-3 mediated suppression of EBNA-2 transactivation and LCL growth maintenance are directly linked to each other remain an open question. This is only can be answered when the complete molecular profile of these protein complexes would be identified in near future.

### Transcriptional activation—intrinsic activity and structural resemblance with bZIP domain

In addition to transcriptional repression, EBNA-3C can also function as a transcriptional activator. For example, EBNA-3C mediated transactivation of LMP-1 promoter, although this activity was seemed to be independent of RBP-Jk interaction. Initial experiments using EBNA-3 proteins in order to unravel its effect on phenotypic changes in B-cell surface markers led to the discovery of EBNA-3C induced CD21 expression (Allday et al., 1993). The activation domain of EBNA-3C was mapped within the C-terminal aa 724-826, which shares a sequence homology with the transactivation domain of cellular transcription factor Sp1 (Marshall and Sample, 1995). However, the transactivation ability was not as strong as its repression. EBNA-3C was further recognized as a strong transcriptional regulator by showing interaction with a TATA-box binding protein, or containing a basic leucine zipper (bZIP) sequence located within the N-terminal residues 244-291 (West, 2006). However, the EBNA-3C bZIP domain portrays a non-canonical bZIP sequence without any appropriate sequence homology or structural resemblance with any known cellular bZIP domains, apart from a tandem repeats of four leucine residues (Amoutzias et al., 2007). Perhaps the lack of DNA binding ability of EBNA-3C corroborates the lack of proper bZIP sequence homology. Further in depth analyses of this bZIP domain through typical biochemical methods such as circular dichroism (CD) spectroscopy and analytical ultracentrifugation imply that this domain does not form stable coiled-coil structures or promote dimerization, which are the common characteristics among well-known leucine zipper domains of DNA binding transcription factors (West, 2006; Amoutzias et al., 2007). On the other hand, substitution of the repeated four leucine residues with prolines resulted in dramatic reduction of RBP-Jk binding and also resulted in a change in the length of the predicted helical structure of the zipper. Likewise, modifications in the charged residues of the basic portion of the EBNA-3C bZIP domain inhibited the RBP-Jk interaction and so as EBNA-3C mediated transcriptional repression (West, 2006; Saha and Robertson, 2013). These results suggests that though EBNA-3C contains a non-canonical bZIP sequence which affect the DNA-binding ability, maintaining proper secondary and tertiary structure within this sequence is essential for downstream activity.

### Carboxy-terminal binding protein (CtBP)

CtBP, initially identified as adenovirus E1A Carboxy-terminal Binding Protein, is a transcriptional co-repressor that can adapt a transcriptionally active chromatin into a transcriptionally silent state (Chinnadurai, 2002, 2009). CtBP is now referred as two closely related transcription factors - CtBP1 and CtBP2 (Chinnadurai, 2002, 2009). Although these two proteins share a substantial aa sequence homology, it is yet to be known that at what extent these proteins are functionally redundant. However, unquestionably both proteins largely function as transcriptional co-repressors and being recruited by factors that have the conserved CtBP-binding ProLeu-Asp-Leu-Ser “PLDLS” motif (Chinnadurai, 2002, 2009). Both EBNA-3A and -3C were shown to strongly interact with CtBP1 via the CtBP binding motifs. As both EBNA-3C and EBNA-3A interact with CtBP1 via the typical CtBP binding motif (Touitou et al., 2001; Hickabottom et al., 2002), it is conceivable that they also can form complex with CtBP2. However, to the best of our knowledge this has not yet been verified. The initial functional relevance of this conserved motif was determined using simple reporter assays. In contrast to the wild-type EBNA-3C, deletion mutant amino acid residues 728-732 “PLDLS” fused with Gal4 DNA binding domains resulted in activation of a CAT reporter, suggesting that CtBP1 might play a critical role in attenuating the EBNA-3C transactivation domain at the C-terminal region (Touitou et al., 2001). Moreover, alteration of this conserved region also impaired the transforming ability of EBNA-3C in cooperation with oncogenic Ha-ras (Saha and Robertson, 2013). In contrast to EBNA-3C, EBNA-3A contains two non-consensus bipartite CtBP binding motifs located at the C-terminal region spanning aa 857-861 “ALDLS” and 886-890 “VLDLS” (Hickabottom et al., 2002). As similar to EBNA-3C, these binding residues of EBNA-3A were found to be critical for both transcriptional repression as well as transforming ability (Hickabottom et al., 2002). Nevertheless, the precise role of CtBP1 in EBNA-3 regulated B-cell transformation process became more evident when analysis of p16^INK4a^ expression in LCLs established by using three individual CtBP-binding motif mutant viruses for EBNA-3A, 3C and for both -3A and -3C revealed that CtBP recruitment is absolutely critical in the EBNA-3A and -3C-mediated epigenetic repression of the p16^INK4a^ promoter (Skalska et al., 2010). However, interestingly no EBNA-3A or -3C/CtBP complexes have been demonstrated on the p16^INK4a^ promoter, there are consistent reports that showed that the CtBP-binding motif is typically essential for LCL growth maintenance using conditional EBNA-3C-expressing LCLs and subsequent rescue studies (Lee et al., 2009).

In contrast, a recent investigation using ChIP-Seq and ChIP-qPCR techniques demonstrated that EBNA3-enriched sites were specifically located in CtBP2 locus instead of CtBP1 locus (McClellan et al., 2013). Interestingly, earlier results from different group demonstrated that only CtBP2 expression is negatively controlled by EBNA-3A (Hertle et al., 2009). However, EBNA-3B and -3C had no effect on CtBP2 expression in corresponding expression-deficient LCLs (White et al., 2010; Skalska et al., 2013). In contrast to CtBP2, similar investigation on CtBP1 expression demonstrated a relatively higher expression level in all EBV-infected cells irrespective of EBNA-3 expression (White et al., 2010; Skalska et al., 2013), signifying a differential regulation pattern of CtBP1 and CtBP2 in response to EBV infection. Consistent with EBNA-3A mediated repression of CtBP2 locus, it has been further demonstrated that only EBNA-3A but not the other bound EBNA-3 proteins including EBNA-2 blocked chromatin looping of the enhancer sequence required for transcriptional initiation (McClellan et al., 2013).

### DP103/Gemin3 and survival of motor neurons (SMN) protein—possible role in RNA processing

Gemin3 (also named as DDX20 or DP103), a member of ATP-dependent RNA helicase family plays several important roles in RNA metabolism (Yan et al., 2003; Fuller-Pace et al., 2007). This family of proteins contain several conserved motifs including the “ASP-Glu-Ala-Asp” or “DEAD box” motif (Yan et al., 2003). The protein along with survival of motor neurons (SMN) complex was initially identified as cellular interacting partners of both EBNA-2 and EBNA-3C using a yeast-2 hybrid screening (Grundhoff et al., 1999). The binding domain of EBNA-3C was mapped within the C-terminal aa 534-778 (Grundhoff et al., 1999). Gemin3 was shown to play a role in gene transcription regulation, through interacting with a number of cellular transcription factors including steroidogenic factor 1 (SF-1), early growth response protein 2 (Egr2), forkhead transcription factor FOXL2 and mitogen Ets repressor METS (Cai et al., 2011; Saha and Robertson, 2013). However, the precise role of Gemin3 in EBV transformed B-lymphocytes was still in debate. Interestingly, Gemin3 was simultaneously identified as a component of SMN complex, which plays an important role in small nuclear ribonucleoproteins (snRNP) proteins assembly (Grundhoff et al., 1999; Battle et al., 2006). The speckled fashion of nuclear expression pattern of EBNA-3 proteins appeared to be stable throughout the cell-cycle. GFP-tagged expression constructs encoding EBNA-3C truncations identified aa 733-808 as the mediator of this nuclear localization pattern. Remarkably, both EBNA-3C and EBNA-3A appeared to co-localize to the identical granular structures, and subsequent analysis demonstrated that EBNA-3C but not EBNA-3A associates with SMN complex in these granules (Krauer K. et al., 2004; Krauer, K. G. et al., 2004a), suggesting possible involvement of EBNA-3C in RNA processing. However, to date no such phenomena has been established for EBNA-3 proteins. Interestingly, a different role for Gemin3 has recently been demonstrated in connection to EBNA-3C mediated transcriptional activity. EBNA-3C induces Gemin3 accumulation in EBV-transformed primary B-lymphocytes and stabilizes a complex formation with p53 tumor suppressor where it acts as a negative regulator through attenuating p53 transcription and apoptotic activities (Cai et al., 2011).

### EBNA-3 proteins target c-Myc transcriptional activity *in vitro*

Both c-Myc translocation and EBV infection status concurrently play critical roles in the development of Burkitt's lymphoma (BL) (Brady et al., 2007). Besides its direct role in transformation, c-Myc can also promote double-stranded DNA breaks and chromosomal aberrations and thereby inducing apoptosis utilizing ATM/Chk2/p53 signaling cascade (Hoffman and Liebermann, 2008). In general, BL expresses a restricted latency I program with only EBNA-1 expression (Molyneux et al., 2012). However, it has been suggested that a subset of *in vitro* generated BL clones may retain the expression of EBNA-3 proteins in either a Wp-restricted latency program with EBNA-2 deletion or a rare EBNA-2(+)/LMP-1(−) latency associated program, indicating the importance of EBNA-3 proteins, in particular EBNA-3A and -3C, in regulating BL pathogenesis thorough blocking cellular apoptosis (Kelly et al., 2005, 2006; Anderton et al., 2008). While EBNA-2 accelerates c-Myc transcription, EBNA-3A downregulates c-Myc expression through recruiting RBP-Jk activity (Kaiser et al., 1999; Cooper et al., 2003). Later, EBNA-3C was independently shown to deregulate c-Myc transcription in a luciferase based reporter assay using a c-Myc responsive telomerase reverse transcriptase promoter (Bajaj et al., 2008). In this study, EBNA-3C mediated c-Myc transactivation could be influenced by either a direct protein-protein interaction or enhancing its protein stability through blocking ubiquitin-proteasomal degradation (Bajaj et al., 2008). Interestingly, the EBNA-3C binding domain was mapped at the N-terminal region flanking aa 130-190, responsible to recruit SCF^Skp2^ E3 ligase activity (Bajaj et al., 2008). Since, c-Myc employs Skp2 as its transcriptional cofactor (von der Lehr et al., 2003); it is tempting to speculate that EBNA-3C may also alter its binding capacity toward Skp2 in order to regulate c-Myc transcription. However, as of now there is no direct evidence that confirms EBNA-3 mediated c-Myc deregulation in BL-biopsy samples.

### Role of chromatin remodeling factors in EBNA-3 mediated transcriptional activities

Using Chromatin immunoprecipition (ChIP) and ChIP-Seq techniques, it is now known that EBNA-3 proteins either individually or in group (particularly EBNA-3A and -3C together) extensively modulate more than 1000 cellular genes throughout the genome via recruiting chromatin remodeling activities (Skalska et al., 2010; White et al., 2010; Allday et al., 2015).

The idea has begun with an initial yeast-two hybrid study with the C-terminal domain of EBNA-3C revealed an interaction with a highly conserved histone H1 interacting protein, prothymosin α (Pro-α), involved in cancer propagation through chromatin remodeling and subsequently alter gene transcription (Subramanian et al., 2002a; Ioannou et al., 2012). The Pro-α binding domain of EBNA-3C was mapped within aa 366-393. Interestingly, EBNA-3C was shown to compete with Pro-α for binding with p300, a transcriptional activator with histone acetyltransferase (HAT) activity (Subramanian et al., 2002a; Iyer et al., 2004). Interestingly, both EBNA-3C and Pro-α were shown to interact with p300 at two distinct sites including the CH1 N-terminal region and the bromodomain comprising of CH3 and HAT domains (Subramanian et al., 2002a). Two distinct interacting domains of EBNA-3C were identified—one at the N-terminal region containing both RBP-Jk binding and bZIP domains, and the other at the C-terminal region containing proline and glutamine rich domain required for transcriptional activation. While Pro-α along with p300 activate transcription using a GAL4 DNA binding domain fused promoter assay, the overall activity is down-regulated in the presence of EBNA-3C (Subramanian et al., 2002a).

EBNA-3C was also shown to interact with histone deacetylases - both HDAC1 and HDAC2, suggesting an additional role in transcriptional repression as addition of an HDAC inhibitor rescued EBNA-3C mediated transcriptional suppression of viral Cp promoter (Knight et al., 2003). Interestingly, the HDAC binding domain of EBNA-3C was found to be overlapped with RBP-Jk interacting region spanning aa 1-211. Moreover, EBNA-3C was shown to form stable transcription repression complex containing mSin3A and NcoR1 directly interacting with Pro-α (Knight et al., 2003). Overall, the results indicate that EBNA-3C recruits HDAC activity embedded within a large protein complex comprising several important transcription factors including RBP-Jk for transcriptional repression—such as from Cp promoter.

In addition to modifying HAT and HDAC activities, EBNA-3 proteins precisely EBNA-3A and -3C were also shown to recruit DNA-methyltransferase (DNMT) activity in order to suppress Bim (BCL2L11) tumor suppressor expression in Burkitt's lymphoma cells (Anderton et al., 2008; Paschos et al., 2009) (discussed in more details in later section). Later, ChIP analyses demonstrated that EBV infection leads to the recruitment of polycomb repressive complex (PRC)2 core subunits and the trimethylation of histone H3 lysine 27 (H3K27me3) at the Bim locus resulted in transcriptional repression. It has been suggested that EBV infection is essential to recruit histone methyl transferases both SUZ12 and EZH2 to establish functional PRC2. Since formation of PRC2 complex at the Bim locus appeared to be reliant on both EBNA-3A and -3C expression, It has been suggested that EBNA-3 proteins might directly interact with PRC2 (Paschos et al., 2012; McClellan et al., 2013; Jiang et al., 2014). However, the precise molecular mechanism by which EBV latent proteins recruit polycomb complexes to restrain Bim expression is yet to be determined.

Both EBNA-3A and -3C were shown to downregulate expression levels of multiple Cyclin dependent kinase inhibitors (CDKI), such as p14^ARF^, p15^INK4a^, p16^INK4a^ via epigenetic regulation (Skalska et al., 2010, 2013). While suppression of p16^INK4a^ expression revealed to play a central role in EBV induced B-cell transformation process (Maruo et al., 2006, 2011; Skalska et al., 2010, 2013), precise roles of p15^INK4b^ and p14^ARF^ in connection to the inhibition of B-cell transformation or LCLs outgrowth, remain largely unclear. Using ChIP analyses on genetically engineered LCLs expressing conditionally active EBNA-3A and -3C, it has been clearly demonstrated that both EBNA-3A and -3C repress p16^INK4a^ expression by recruiting a repressive H3K27me3 epigenetic mark on corresponding CDKN2A locus (Skalska et al., 2010, 2013; Maruo et al., 2011). In addition, recruitment of CtBP1 by EBNA-3A and -3C were also shown to be important for efficient deposition of H3K27me3 on p16^INK4a^ gene locus for its repression (Skalska et al., 2010). Similarly, EBNA-3A was shown to repress p15^INK4a^ *gene* (CDKN2B) transcription through recruiting MIZ1 and H3K27me3 repressive histone modification (Bazot et al., 2014).

## Cell-cycle regulation

### EBNA-3 proteins extensively modulate cell-cycle machinery

The role of the EBNA-3 proteins on cell-cycle regulation was initially demonstrated using an EBV positive Burkitt's lymphoma derived cell line—Raji, where the EBNA-3C gene was deleted (Allday et al., 1993; Allday and Farrell, 1994). Raji (ΔEBNA-3C) cells at high density could be arrested in the G1 phase of the cell-cycle, whereas the cell-cycle activity can be restored by EBNA-3C expression (Allday and Farrell, 1994). Precisely, EBNA-3C was shown to induce LMP-1 transcription and pRb phosphorylation (Allday and Farrell, 1994). In agreement with pRb hyperphosphorylation, EBNA-3C was also shown to transactivate an E2F responsive promoter B-*myb* (Parker et al., 1996). These results signified for the first time that one of the EBNA-3 proteins might play a prominent role in G1-S phase transition of the cell-cycle by targeting the putative pRb-E2F complex. Similarly, EBNA-3C expression in NIH3T3 and U2OS cells rescued the growth arrest at G1 phase caused by serum starvation. Moreover, EBNA-3C was shown to downregulate one of the cyclin dependent kinase inhibitor (CDKI) p27^KIP1^ expression and suppress the pro-metaphase arrest. Collectively, results portrayed a model where EBNA-3C expression led to a complete disruption of multiple cell-cycle checkpoints at both G1/S and G2/M (Parker et al., 2000). In a series of studies we and others have demonstrated that EBNA-3C can physically interact with a number of important proteins involved in cell-cycle regulation at both G1/S and G2/M checkpoints, such as tumor suppressor proteins—pRb and p53, E3-ubiquitin ligase—SCF^Skp2^, oncoproteins—c-Myc, cyclin A, cyclin D1, p53 regulatory proteins—Mdm2, ING4, and ING5, DNA damage responder—E2F1, Chk2, H2AX, and Aurora kinase B among many others (Jha et al., 2013, 2014; Saha and Robertson, 2013).

**Figure 1:**
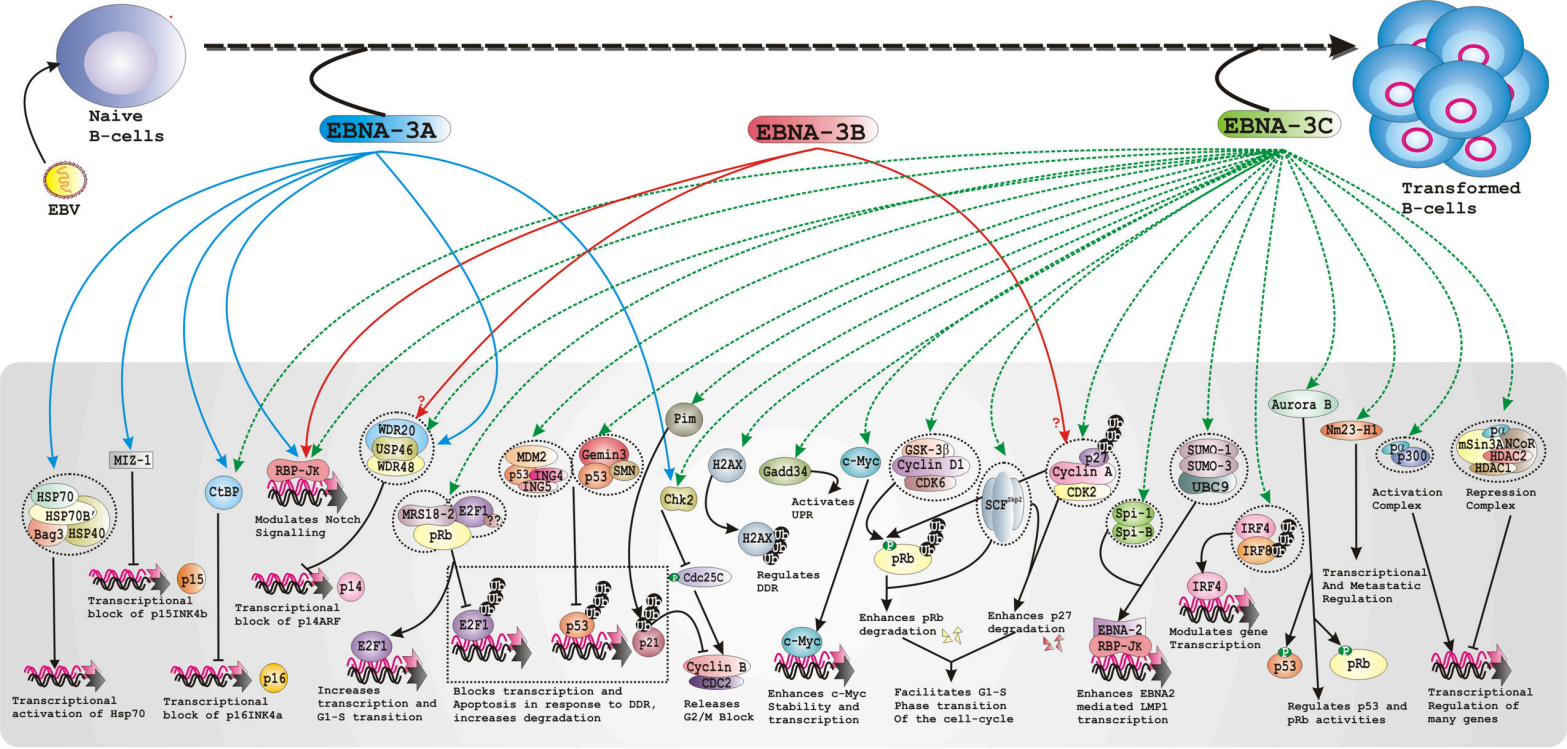
**Schematic represents multiple functions of the EBNA-3 family of proteins in developing EBV associated B-cell lymphomas**.

As earlier discussed that EBNA-3A and -3C but not -3B are essential for B-cell transformation *in vitro*, it was still not clear whether EBNA-3 proteins are important in maintaining LCLs growth once transformed. Using LCLs with conditionally expressed EBNA-3 proteins and trans-complementation assays it has been clearly demonstrated both EBNA-3A and -3C are indispensible for LCLs survival (Maruo et al., 2005, 2006). Using this system it has been determined that EBNA-3C specifically targeted CDK inhibitor p16^INK4A^ (Maruo et al., 2011). Using the similar conditional EBNA-3A and -3C expressing LCL models, it has been further confirmed that RBP-Jk binding and CtBP motifs are important for maintaining LCL growth (Maruo et al., 2005, 2009). In the similar study, it has been suggested that the N-terminal residues EBNA-3C that have been shown to recruit various important cell-cycle proteins—SCF^Skp2^, pRb, p53, Mdm2, E2F1, Cyclin A, Cyclin D1 among others are important for maintaining LCLs growth (Maruo et al., 2009). In a recent report, EBNA-3A was shown to interact with a Myc-interacting zinc-finger protein-1 (MIZ1) and that is required for down-regulation of the CDK inhibitor p15^INK4b^ in LCLs (Bazot et al., 2014), suggesting a possible role in EBV-infected B-cell proliferation. Taken together, it is seemingly evident that both EBAN-3A and -3C proteins regulate B-cell transformation and subsequently induce B-cell lymphoma development through targeting major cell-cycle regulators including cyclin-cyclin dependent kinase (CDK) complexes, CDK inhibitors (CDKI), and checkpoint regulators (Figure 1).

### EBNA-3C interacts with all major cyclins

Cell-cycle progression is dependent on the activity of cyclins, a family of proteins whose levels oscillate in synchrony with cell-cycle progression, and its functional partner CDKs (Hochegger et al., 2008). An initial yeast-two hybrid study using C-terminal aa 890-992 of EBNA-3C revealed an interaction with Cyclin A, which was subsequently confirmed by both *in vitro* and *in vivo* binding experiments (Knight and Robertson, 2004). EBNA-3C was also shown to block CDKI p27^Kip1^ actions on Cyclin A/CDK2 regulated kinase activity, suggesting a potential mechanism for facilitating G1/S transition of the cell-cycle (Knight and Robertson, 2004). As CDK2 form complexes with both Cyclin A and Cyclin E during the G1 to S phases of the cell-cycle, it was further confirmed that the effect was specific for Cyclin A, as co-expression of Cyclin E/CDK2 with EBNA-3C was not able to retrieve p27-mediated inhibition. However, EBNA-3C could physically interact with all the cyclins including Cyclin A, D1 and E in an *in vitro* interaction study. Interestingly, although the Cyclin A binding domain was initially mapped at extreme C-terminal region amino acid residues 957-990, following studies revealed a stronger interaction domain at the N-terminal region amino acid residues 130-159, surrounding the conserved homology domain of the EBNA-3 proteins (Knight et al., 2004). Indeed, in an *in vitro* study EBNA-3B but surprisingly not EBNA-3A demonstrated a modest binding activity (Knight et al., 2004). In addition, the N-terminal binding domain of EBNA-3C was appeared to be responsible for restricting p27^Kip1^-mediated inhibition of Cyclin A/CDK2 kinasing activity, whereas C-terminal binding domain perhaps plays as a stabilizing element of this complex (Knight and Robertson, 2004; Knight et al., 2004).

In a recent work it has been shown that EBNA-3C can form a stable complex with Cyclin D1/CDK6 in both stably expressing as well as EBV-transformed B-cells. Whether the interaction between EBNA-3C with different cyclins is dependent on different cell-cycle stages and how these interactions eventually trigger EBNA-3C mediated B-cell transformation, is remain unexplored. It has been proposed that Cyclin D proteins (D1, D2, and D3) besides initiating cell-cycle at G1 phase, may have distinct biological activities at specific steps of B-cell differentiation, and their expression can be differentially regulated in response to EBV-infection (Palmero et al., 1993; Saha et al., 2011b). Indeed, in contrast to the previously published results, *in vitro* EBV infection in primary B-lymphocytes along with EBV-positive BL derived cell lines resulted in significant up-regulation of all the D-type cyclins (Palmero et al., 1993; Saha et al., 2011b, 2015). Elevation of Cyclin D1 protein level without affecting its genetic structure, has been shown to be one of the potential mechanisms for its deregulated activities at G1-S phase transition of the cell-cycle (Musgrove et al., 2011). Moreover, Cyclin D1 expression is strictly cell-cycle dependent and its expression is regulated by both sub-cellular localization and ubiquitin-targeted proteosomal machineries (Musgrove et al., 2011). During G1-S transition of the cell-cycle, Cyclin D1 appeared to be more nuclear localized with reduced proteolytic activity and inhibited GSK-3β kinasing function which phosphorylates at T_286_ cyclin D1 leading to cycltoplasmic localization for ubiquitin-proteasome mediated degradation (Alao, 2007; Musgrove et al., 2011). Remarkably, EBNA-3C was shown to play a dual role that leads to an increasing nuclear localization of Cyclin D1 by blocking its poly-ubiquitination level assisted with inhibition of GSK-3β mediated phosphorylation (Saha et al., 2011b). Given that overexpression of Cyclin D1 is directly linked to development of cancer development through integrating the control of pRb phosphorylation with the transcriptional activity of E2F transcription factors, targeting Cyclin D1 degradation would offer as a potential therapeutic mechanism in EBV associated B-cell lymphomas, where EBNA-3C is expressed.

### Targeting pRb provides a best possible link to facilitate G1 to S phase transition of the cell-cycle

As discussed earlier, the initial clue for a possible interaction between EBNA-3C and pRb came from the observation that ectopic EBNA-3C expression can rescue Raji cells arrested at G1 phase through increasing the pRb phosphorylation (Allday and Farrell, 1994). In addition, EBNA-3C as similar to other tumor virus encoded oncoproteins such as adenovirus E1A and HPV E7 was shown to interact *in vitro* with pRb and regulate its downstream activities through E2F transcription factors (Parker et al., 1996). Moreover, all these viral oncoproteins contain a pRb interactive motif LxCxE (Parker et al., 1996). This led researchers to further investigate on both upstream regulators such as CDK inhibitors and downstream effectors such as E2F mediated transactivation process. Later, EBNA-3C was shown to form a complex with pRb in cell (Knight et al., 2005a; Kashuba et al., 2008), in which a mitochondrial ribosomal protein S18-2 (MRPS18-2) acts as a bridging protein between EBNA-3C and pRb and plays an important role in deregulating its downstream activities (Kashuba et al., 2008). Interestingly, the binding between pRb and EBNA-3C was shown to be stabilized in the presence of proteasomal inhibitor (Knight et al., 2005a), indicates that EBNA-3C might also be involved in pRb degradation besides regulating its phosphorylation status. Indeed, EBNA-3C was shown to enhance pRb polyubiquitination through recruitment of SCF^Skp2^ complex E3 ligase in an *in vitro* setting (Knight et al., 2005a). The pRb interacting domain was mapped at aa 130-159, where aa 140-149 was shown to be important for facilitating pRb degradation (Knight et al., 2005a). In contrast, studies using LCLs with conditionally active EBNA-3C expression, it has been shown that EBNA-3C was not responsible for pRb degradation rather it maintains a hyperphosphorylation status of pRb (Maruo et al., 2006; Zhao et al., 2010). As noted earlier, EBNA-3C can enhance the kinase activities of both G-phase cyclin–Cyclin D1/CDK6 and S-phase cyclin–Cyclin A/CDK2 complexes, which altogether phosphorylate pRb (Knight et al., 2004; Saha et al., 2011b). It has been suggested that EBNA-3C mediated phosphyrylation of pRb act as a prerequisite for accelerating G1-S phase transition of the cell-cycle.

### Chk2: effect of EBNA-3 proteins at G2/M phase

In general, EBV-negative BL derived cell lines are more susceptible to genotoxic agents in comparison to EBV positive lines including LCLs and thereby experiencing cell-cycle arrest at the G2/M checkpoint (Wade and Allday, 2000). In addition, BL derived cell lines stably expressing individual EBNA-3 family proteins but not EBNA-2 or EBNA-LP could also bypass cell-cycle arrest at the G2/M checkpoint (Parker et al., 2000). This led researchers to further study on plausible involvement of ATM/ATR kinase family of proteins in G2/M arrest. In fact, in two separate studies, both EBNA-3A and -3C were demonstrated to form stable complexes with Chk2 protein, a downstream checkpoint kinase of ATM mediated DNA damage response pathway (Krauer K. G. et al., 2004b; Choudhuri et al., 2007). The interaction between EBNA-3C and Chk2 eventually resulted in a phosphorylation of Cdc25c at S_216_, which subsequently promotes its own sequestration in the cytoplasm through interaction with 14-3-3 and in so doing allows the kinase activation of Cyclin B/Cdc2 complex and helps in circumventing the G2/M block induced by drug nocodazole (Choudhuri et al., 2007). This study was the first attempt in order to demonstrate a underlying mechanism by which EBNA-3C disrupts the G2/M checkpoint signaling to maintain the continuous proliferation of EBV-transformed B-cells (Choudhuri et al., 2007). In agreement with this finding, a recent study also showed that EBNA-3C expression is absolutely required to attenuate ATM-Chk2 mediated DNA damage responsive signaling for B-cell transformation (Nikitin et al., 2010; Li and Hayward, 2011).

## Inhibition of apoptotic machineries

Cancer cells sustain mutations in many important cellular components that disrupt normal mechanisms controlling proliferation. Remarkably, both pRb–E2F1 and Mdm2–p53 signaling network are found to be defective in most tumors, emphasizing the central role of these cascades in regulating cell-cycle progression. Two apoptotic pathways exist in mammals - intrinsic and extrinsic, ultimately converge with the activation of cysteine proteases named caspases (Elmore, 2007; Jendrossek, 2012). Interestingly, EBNA-3 proteins in particular EBNA-3C was shown to extensively regulate many tumor suppressor proteins via intrinsic apoptotic pathway (Figure 1).

### EBNA-3C regulates p53-induced apoptosis by multiple mechanisms

The p53 tumor suppressor is perhaps represents one of the most important drug targets in cancer therapy as the corresponding gene has been found to be mutated or deleted in half of all malignant tumors, whereas the other half express wild-type p53 and in one way or another is functionally blocked (Essmann and Schulze-Osthoff, 2012; Mirzayans et al., 2012). As similar to pRb, tumor viruses have evolved multiple strategies to inhibit directly p53 functions or its upstream or downstream effectors are being inactivated indirectly affecting p53 mediated transcriptional as well as apoptotic activities (Saha et al., 2010). As similar to many other tumor virus encoded antigens, EBNA-3C was also shown to attenuate p53-induced apoptosis employing multiple mechanisms (Saha and Robertson, 2013). EBNA-3C forms a complex with p53 and the interacting domain was mapped near the N-terminal region comprising aa 130-190 (Yi et al., 2009). Interestingly, these binding residues, as described in the earlier section, have been previously shown to interact with several other important cell-cycle proteins, such as SCF^Skp2^, pRb, c-Myc, Cyclin A, Cyclin E, Cyclin D1, and RBP-Jκ, signifying its critical involvement in cell-cycle deregulation (Robertson et al., 1996; Knight et al., 2005a,b; Bajaj et al., 2008; Saha et al., 2011b; Saha and Robertson, 2013). Additionally, genetic study using recombinant EBV virus expressing conditionally active EBNA-3C revealed that absence of this particular domain was unable to maintain LCLs growth (Maruo et al., 2009). Importantly, EBNA-3C interacts with p53 with its central DNA-binding and C-terminal oligomerization domains (Yi et al., 2009), providing clues that EBNA-3C might regulate its transcription activities. Indeed, reporter assays using p53 responsive promoter element demonstrated that EBNA-3C appreciably inhibits p53 transactivation and subsequent apoptotic activities (Yi et al., 2009). Since p53 regulated transcription can be modulated by several means, it has been speculated that EBNA-3C could employ other potential mechanisms.

In order to investigate other potential mechanisms by which EBNA-3C could affect p53 mediated transcription and as a result apoptotic regulation, EBNA-3C was shown to interact with p53 modulators ING4 and ING5 belong to ING (inhibitor of growth) family (Saha et al., 2011a). The best known function of ING proteins, whose expression was shown to be significantly reduced in many cancer types, is their cooperation with p53 in tumor suppression (Russell et al., 2006; Jafarnejad and Li, 2011). EBNA-3C interacts with both ING4 and ING5 in a p53 independent manner as binding studies were performed in p53 positive *in vitro* EBV transformed LCLs, EBNA-3C stably expressing BL derived cell line BJAB where p53 is genetically defective and ectopic expression systems in Saos-2 where p53 is deleted (Saha et al., 2011a). However, the binding region of EBNA-3C was shown to be overlapped with p53 interacting site at the N-terminal region covering aa 129-200 (Saha et al., 2011a), suggesting that interaction with ING-proteins may influence its binding affinity toward p53. In fact, increasing dose of p53 concentration appreciably impeded the complex formation between EBNA-3C with ING proteins (Saha et al., 2011a). Additionally, the binding domains for both EBNA-3C and p53 were mapped at the identical residues of ING proteins—comprising the bipartite nuclear localization domain (NLS1 and NLS2) of ING4 and the conserved PHD domain of ING5 (Saha et al., 2011a). The PHD domain through recruiting HAT and HDAC activities represents as a central structural identity of ING proteins (Russell et al., 2006). However, whether EBNA-3C and/or p53 modulate its chromatin remodeling functions remains unclear. Similarly the interaction of ING4 NLS domain with EBNA-3C and p53 affects its sub-cellular localization is still speculative. Nonetheless, EBNA-3C substantially antagonizes ING4 and ING5 promoted p53 mediated ant-proliferative activities possibly through blocking the interaction of ING proteins with p53 (Saha et al., 2011a) and thus restoration of ING functions in order to activate p53 induced apoptosis offers a potential therapeutic approach against EBV associated B-cell lymphomas.

The ubiquitin-mediated proteasomal degradation of p53 by Mdm2, one of its many negative regulators, symbolizes as one of the most important regulations in p53 mediated tumor suppressive activities (Di et al., 2011; Essmann and Schulze-Osthoff, 2012). It has been shown that *in vitro* EBV transformed LCLs expressing wild-type p53 are sensitive to Nutlin-3a mediated growth suppression, which exclusively targets p53-Mdm2 interaction and thereby increasing p53 stability and apoptosis (Forte and Luftig, 2009). In parallel with this finding, EBNA-3C was shown to recruit Mdm2 E3 ligase activity in order to facilitate p53 degradation (Saha et al., 2009). As discussed earlier, EBNA-3C efficiently deregulates the ubiquitin-proteasome machinery and that profoundly affects stability of many tumor suppressor proteins—p27^Kip1^ and pRb through enhancing their degradation as well as increases the stability of products of several proto-oncogenes such as Cyclin D1 and c-Myc (Knight et al., 2005a,b; Bajaj et al., 2008; Saha et al., 2011b). In this study, the authors evidently demonstrated that other than regulating its own polyubiquitination, EBNA-3C can also obstruct Mdm2-ployubiquitination and thereby increasing its stability as similar to Cyclin D1 and c-Myc (Bajaj et al., 2008; Saha et al., 2009, 2011b). In addition to negatively affecting p53 transcription and apoptotic activity, Mdm2 can also deregulate apoptotic function of pRb and E2F1, which were also shown to interact with EBNA-3C (Knight et al., 2005a; Polager and Ginsberg, 2009; Saha et al., 2012). However, so far there is no direct evidence that shows EBNA-3C recruits Mdm2 activity in order to control the pRb-E2F1 arm at the G1-S phase of the cell-cycle. Interestingly, EBNA-3C utilizes the similar N-terminal domain comprising aa 130-190 formerly identified as interacting region of many important cell-cycle regulators including p53, in order to recruit Mdm2 E3 ligase activity toward p53 degradation (Saha et al., 2009). In addition, the central acidic domain of Mdm2 was shown to be responsible for interaction with EBNA-3C (Saha et al., 2009). Interestingly, a number of earlier studies revealed that this central acidic domain plays an important role in regulating Mdm2 mediated E3 ligase activity toward p53 degradation through interacting with many positive such as p14^ARF^ and pRb as well as negative regulators including p300 (Kawai et al., 2003). Collectively, this study along with the study with Nutlin-3a implicated a potential therapeutic mechanism through targeting p53-Mdm2 complex against many EBV associated B-cell lymphomas expressing functionally active p53.

As discussed in the previous section, the interaction between EBNA-3C and DP103/Gemin3 (belongs to DEAD-box RNA helicase family) has been implicated in p53 mediated transcriptional as well as apoptotic activities (Yan et al., 2003; Cai et al., 2011). In agreement with previously described EBNA-3C regulated p53 activities, this work further appended as a potential mechanism through which p53 is deregulated in EBV associated B-cell lymphomas. Although Gemin3 has previously been characterized as a transcriptional repressor, the precise mechanism by which Gemin3 regulates transcriptional activity is not completely known beside its role in RNA metabolism (Yan et al., 2003). Since, Gemin3 through recruiting HDAC and sumo activities represses transcriptional activation, it has been suggested that EBNA-3C/Gemin3 complex can also alter p53-dependent anti-proliferative activities by affecting its acetylation and sumoylation status. Given that the critical role of Gemin3 in cancer development, prompted us to speculate that high throughput screening of helicase inhibitors would result in prospective therapeutic strategy against EBV associated B-cell lymphomas possibly through initiating p53 regulated apoptosis.

In a recent study, EBNA-3C was additionally shown to block p53 expression and subsequent transcription and apoptotic activities through stabilizing and recruiting Aurora kinase B mediated kinase activity (Jha et al., 2013, 2015).

### EBNA-3C antagonizes E2F1-regulated apoptosis in response to DNA damage signals

As discussed in earlier sections, EBNA-3C targets a number of upstream components of the E2F1 signaling pathway involved in cell-cycle, DNA repair, differentiation as well as apoptosis in both p53 dependent and independent manner (Krauer K. G. et al., 2004b; Knight et al., 2005a; Saha et al., 2011b). The interaction between EBNA-3C and pRb led further investigation to determine whether EBNA-3C can also form a complex with its downstream regulator, E2F1 and thereby facilitating the G1-S transition of the cell-cycle. Indeed, EBNA-3C was shown to physically interact with E2F1 (Saha et al., 2012), however in a pRb independent manner, suggesting that EBNA-3C may regulate E2F1 activity through a different mechanism. Since p53 has been found to be mutated or functionally deactivated in most of the cancers, targeting E2F1-mediated apoptosis in response to DNA damage signals can provide as an additional therapeutic means (Polager and Ginsberg, 2009; Wu and Yu, 2009). In accordance with this notion, EBNA-3C was shown to inhibit E2F1 dependent apoptotic activities through targeting its downstream apoptotic regulators such as p73 and Apaf-1 in EBV positive B-cells (Saha et al., 2012). In this connection, another group has recently shown that EBNA-3C forms a stable complex with p73 and blocks doxirubicin induced p73 mediated apoptosis (Sahu et al., 2014). The N-terminal DNA binding domain of E2F1 aa 1-243, liable to apoptotic regulation, binds to two discrete regions of EBNA-3C, positioned at N-terminal aa 100-200 and C-terminal aa 621-700 (Saha et al., 2012). Importantly, while the N-terminal binding domain of EBNA-3C previously shown to interact with many other cell-cycle proteins as discussed earlier directly interacts with E2F1, the C-terminal region requires unidentified cellular protein(s) to form a complex with E2F1 (Saha et al., 2012). Moreover, using trans-complementation assay in LCLs expressing conditionally active EBNA-3C, this N-terminal binding domain but not the C-terminal region of EBNA-3C was shown to be critical in maintaining LCLs growth (Maruo et al., 2009). Another group demonstrated that EBNA-3C is absolutely essential in attenuating DNA damage response induced during early stage of viral infection of primary B-lymphocytes in order to facilitate B-cell transformation (Nikitin et al., 2010). In harmony with this finding, EBNA-3C knockout EBV was also shown to be lacking its ability in restraining E2F1 mediated DNA damage response during the early stages of infection of nascent B-cells (Saha et al., 2012). As similar to p53, DNA damage signals led to an induction and stabilization of E2F1 expression, where both ubiquitin-tagged proteasomal degradation and dissociation from pRb at G1-S phase of the cell-cycle play important roles (Wu and Yu, 2009). Since SCF^Skp2^ as one of the many E3 ligases involved in E2F1 degradation (Harper and Elledge, 1999), it is tempting to speculate that EBNA-3C may also recruit this E3 ligase activity for regulating E2F1 stability and thus transcription. Although it remains elusive that whether EBNA-3C specifically employs SCF^Skp2^ E3 ligase activity, EBNA-3C accelerates E2F1 degradation in an ubiquitin-proteasome dependent manner (Saha et al., 2012). In addition, DNA damage sensor components such as ATM-Chk2 and ATR-Chk1 stabilize E2F1 expression through phosphorylation and thereby regulating E2F1 mediated apoptotic signaling cascade (Wu and Yu, 2009). As previously discussed EBNA-3C mediated deregulation of ATM-Chk2 cascade demands further investigation in controlling E2F1-targeted apoptosis in the context of EBV associated B-cell lymphomas. Overall, these findings suggest E2F1 as a potential therapeutic target regardless of p53 functional status against several EBV associated B-cell lymphomas.

### EBNA-3A and -3C regulate bim-mediated apoptosis through epigenetic regulation

The tumor suppressor protein Bim or *BCL2L11* represents one of the crucial members of Bcl-2 (B-cell lymphoma 2) family that induces apoptosis (Hughes et al., 2006). Bim plays an important role during B-cell lymphomagenesis as deletion of even a single allele can radically increase B-cell lymphoma development in Eμ-Myc transgenic mice with constitutive c-Myc expression within B-cells (Egle et al., 2004; Richter-Larrea et al., 2010). As earlier discussed deregulation of c-Myc activity due to chromosomal translocations is a characteristic feature of Burkitt's lymphoma (BL) and interestingly, Bim regulated apoptosis was also shown to be coupled with c-Myc deregulation that ultimately helps in developing B-cell lymphoma (Richter-Larrea et al., 2010). Analysis of EBV latent gene expression patterns in different EBV-positive BL derived cell lines revealed that the EBNA-3 proteins might play an important role in regulating Bim mediated apoptosis. Indeed, using genetically engineered EBV, lacking individual EBNA-3 ORFs, EBNA-3A and -3C but not -3B blocked Bim mediated apoptosis in response to multiple cytotoxic drugs in a BL derived cell line, providing a possible explanation by which EBV contributes to BL-pathogenesis (Anderton et al., 2008). In addition, Bim expression was drastically increased in response to both EBNA-3A and -3C knockout viruses in contrast to cells infected with either wild-type or mutant virus lacking expression of other EBNA genes including EBNA-3B (Anderton et al., 2008). The suppression of Bim expression in EBV positive B-cells by EBNA-3 proteins was established at transcriptional level via epigenetic modification as treatment with HDAC and DNA-methyltransferase enzymes (DNMT) inhibitors enhanced Bim expression (Anderton et al., 2008). Epigenetic regulations including hypermethylation of cytosine residue at CpG islands and covalent modifications most prominently methylation and acetylation to the N-terminal tails of histones, regulate gene expression through varying the chromatin structure in a heritable manner and thus characterized as a hallmark of cancer development (Bártová et al., 2008; Sandoval and Esteller, 2012). As discussed in the earlier section, EBNA-3C can recruit many chromatin modification enzymes in order to regulate gene transcription (Radkov et al., 1999; Cotter and Robertson, 2000; Knight et al., 2003; Skalska et al., 2010). However, whether EBNA-3A or -3C may also recruit DNMT activity, which is a common mechanism for silencing transcriptional activation of many tumor suppressor genes in most cancers, are unknown. Nevertheless, it has been proposed that EBNA-3A and -3C repress Bim expression and subsequently its apoptosis in BL through histone modification H3K27-Me3 (trimethylation of histone H3 lysine 27) and CpG hypermethylation at the Bim promoter (Paschos et al., 2009). Interestingly, recently using a PCR microarray its has been shown that EBV infection in primary B-lymphocytes led to a global transcriptional repression of an array of tumor suppressor genes through recruiting hypermethylation activity (Saha et al., 2015), suggesting a common mechanism by which EBV promotes B-cell immortalization.

## Role in metastasis

### EBNA-3C regulates Nm23-H1 mediated anti-metastatic activities

Nm23-H1 belongs to nucleoside diphosphate kinases (NDPKs) family, was identified as the first anti-metastatic protein significantly implicated in cancer progression through regulating various signaling pathways (Murakami et al., 2009; Marshall et al., 2010). However there are many conflicting data, an altered Nm23-H1 expression, both at protein and RNA levels coupled with its metastatic activity was shown to directly associate with many cancer types, including tumor virus associated B-cell lymphomas (Marshall et al., 2010; Saha and Robertson, 2011b). Undoubtedly, EBNA-3C represents as one of the best studied Nm23-H1 interacting partners, which was initially identified in a yeast two hybrid screening (Subramanian et al., 2001; Saha and Robertson, 2011b). Using series of truncated EBNA-3C regions in both *in vitro* and *in vivo* settings, the binding site was mapped at aa 657-675 (Subramanian and Robertson, 2002). The interaction of EBNA-3C with Nm23-H1 resulted in salvaging Nm23-H1 induced anti-proliferative effects on cell migration examined in multiple cell lines (Subramanian and Robertson, 2002). Contradictorily, EBNA-3C can also accelerate Nm23-H1 mediated transcriptional activity on multiple promoters - Cox-2, αv-integrin and MMP-9 (Saha and Robertson, 2011b). Remarkably, EBNA-3C co-expression leads to relocation of Nm23-H1's cytoplasmic signal to mostly nucleus, providing a possible explanation of enhanced Nm23-H1 mediated transcriptional activity (Subramanian et al., 2001; Saha and Robertson, 2011b). However, how these changes eventually influence Nm23-H1's anti-metastatic activities is not clear. In this context, an *in vivo* study using nude mice as model system was conducted to determine the magnitude of EBNA-3C's ability in suppressing Nm23-H1 mediated anti-metastatic potential (Kaul et al., 2007). The study demonstrated that EBNA-3C significantly inhibits Nm23-H1 activity and induces the initial tumor formation, while at the later stage both EBNA-3C and Nm23-H1 seemed to have no function regarding tumor progression (Kaul et al., 2007). This study also corroborated with a previous study aimed to analyze Nm23-H1 expression levels in established EBV-positive and negative B-cells with no apparent change, indicating that Nm23-H1 might not be important once the cancer has already developed (Murakami et al., 2009; Saha and Robertson, 2011b). Since genetically engineered EBV lacking EBNA-3C ORF is unable to transform primary B-lymphocytes, EBNA-3C may regulate Nm23-H1 activities at early stage of infection. In fact, a recent study using a PCR microarray showed that EBV infection in nascent peripheral B-lymphocytes led to an increased hypermethylation pattern of Nm23-H1 promoter and thereby affecting its transcription (Saha et al., 2015). However, whether EBAN-3C has any direct role in Nm23-H1 promoter hypermethylation and subsequently expression level is currently under investigation.

Blast analysis of the Nm23-H1 binding residues of EBNA-3C revealed a considerable sequence homology with another metastasis suppressor Necdin, a member of the melanoma-associated antigen (MAGE) family of proteins comprising of more than 60 genes that share the highly conserved MAGE homology domain (MHD) (Kaul et al., 2009). Necdin acts as a transcriptional repressor either by directly bind to the DNA or through its interaction with major transcription regulators such as p53, E2F1, and Hif-1α (Matsumoto et al., 2001). Notably, elevated CpG-methylation of Necdin promoter in EBV transformed B-lymphocytes was observed as compared to nascent B-lymphocytes, suggesting that EBV latent antigens may regulate Necdin expression and subsequently its function (Kaul et al., 2009). In addition, Necdin expression level was shown to be particularly lower in EBV-positive BL cells than that of negative counterpart, further suggesting epigenetic regulation might play an important role in Necdin expression in EBV positive B-cells (Kaul et al., 2009). However, whether EBNA-3C or any other EBV latent antigens recruit epigenetic machineries in order to control Necdin expression is yet to be known. On the other hand, EBNA-3C coupled with Nm23-H1 was shown to deregulate Necdin mediated growth suppression and anti-angiogenic property possibly through affecting at both transcriptional and subcellular localization levels (Kaul et al., 2009; Saha and Robertson, 2011b). Overall, this study suggests a novel role for Necdin in regulating virus-associated human cancers development.

## Conclusion

Over the last decade a significant progress has been made toward our understanding of how the EBNA-3 proteins contribute to the induction and subsequently development of several B-cell lymphomas particularly in an immune-compromised scenario. EBNA-3 proteins—particularly EBNA-3A and -3C deregulate a number of cellular pathways including cell-cycle, apoptosis, and metastasis largely through direct protein-protein interaction. EBNA-3C, in particular regulates an array of cellular protein level through manipulating ubiquitin-targeted proteasomal machinery. In addition, both EBNA-3A and -3C deregulate gene transcription through recruiting several chromatin remodeling factors. Newer technological advancements such as genetically modified viruses, microarray based techniques, and whole transcriptomic analyses have essentially demonstrated that these proteins play a major role in EBV induced B-cell lymphomagenesis and therefore may facilitate in the development of targeted therapeutics in near future.

## Author contributions

SB, SG, PB and AS together wrote the paper. AS conceived the idea and finally edited the manuscript.

### Conflict of interest statement

The authors declare that the research was conducted in the absence of any commercial or financial relationships that could be construed as a potential conflict of interest.
